# Single Layer Centrifugation with Androcoll-P Can Be Scaled-Up to Process Larger Volumes of Boar Semen

**DOI:** 10.5402/2011/548385

**Published:** 2010-11-29

**Authors:** Marjet van Wienen, Anders Johannisson, Margareta Wallgren, Joyce Parlevliet, Jane M. Morrell

**Affiliations:** ^1^Division of Reproduction, Department of Clinical Sciences, Swedish University of Agricultural Sciences—SLU, P. O. Box 7054, 75007 Uppsala, Sweden; ^2^Faculty of Veterinary Medicine, 3508TD Utrecht, The Netherlands; ^3^Institute of Anatomy, Physiology and Biochemistry, Swedish University of Agricultural Sciences, P. O. Box 7011, 75007 Uppsala, Sweden; ^4^Quality Genetics, Hörby, Sweden

## Abstract

The objective of this study was to scale-up the procedure for Single Layer Centrifugation (SLC) through Androcoll^TM^-P, as a preliminary step towords processing the whole ejaculate. The first experiment compared Single Layer Centrifugation using 4.5 mL and 15 mL extended ejaculate (SLC-4.5 and SLC-15, resp.), assessing sperm quality by objective motility analysis, morphology, viability, and the production of reactive oxygen species (ROS). In the second experiment, SLC-4.5 was compared to Single Layer Centrifugation with 25 mL extended ejaculate (SLC-25) using motility analysis and morphology. In both experiments, normal morphology and linear motility were significantly higher in the SLC-selected samples than in the uncentrifuged controls (*P* < .001), whereas total motility and membrane integrity were unchanged. Although ROS production was higher in the SLC-selected samples than in the controls (*P* < .01), this might have been due to the presence of antioxidants in seminal plasma in the latter. In conclusion, there was no difference in sperm quality between SLC-4.5 and SLC-15 samples, or between SLC-4.5 and SLC-25 samples, indicating that the SLC method can be scaled-up successfully.

## 1. Introduction

A technique for improving sperm quality is considered desirable in many domestic species, for example, cattle and horses, where sperm quality is related to pregnancy rates after artificial insemination (AI). In cattle, pregnancy rate is correlated with increasing sperm motility in the sperm dose, whereas in the boar, increasing motility over 60%–70% is not reflected in increased pregnancy rates [1, 2]. Similarly, stallion sperm morphology is highly correlated to pregnancy rate, whereas the correlation is lower in the boar [3]. Selection of boars as breeding sires is usually based on genetics for production characteristics rather than on semen quality, since the high sperm doses used in AI (three billion motile spermatozoa) are thought to compensate for the presence of defective spermatozoa [4, 5]. 

Nevertheless, a method that selects the most robust spermatozoa from an ejaculate could have many advantages for the boar AI industry, either by reducing the sperm number used for AI [5], potentially enhancing sperm survival during cryopreservation, or by removing pathogens in the ejaculate such as bacteria [6] and viruses [7]. Such a selection method would have a cost benefit for the pig industry, depending on the value of the boar, the actual price of semen doses, and the perceived need to increase biosecurity and reduce antibiotic usage.

Single layer centrifugation (SLC) using a silane-coated silica-based formulation optimised for stallion spermatozoa (Androcoll-E) has been shown to select spermatozoa with the best motility, normal morphology, and chromatin integrity [8–10]. Preliminary results with boar spermatozoa indicated that SLC with a boar-specific colloid formulation, Androcoll-P, selects the most highly motile boar spermatozoa [11]. However, the volume used (1.5 mL of a sperm suspension at 100 × 10^6^ spermatozoa/mL) was considered to be impractical for processing large volumes of ejaculate. Recently, the SLC method was scaled up to allow 15–18 mL of stallion ejaculate to be processed through Androcoll-E per 50-mL centrifuge tube [12]. Therefore, the aims of this study were to scale up the SLC procedure through a silane-coated silica colloid (Androcoll-P) to allow larger volumes of extended boar ejaculate to be processed, as a preliminary step for further increases.

## 2. Materials and Methods

Ejaculates were available from 6 mature boars (Swedish Yorkshire, Norwegian Landrace, and Swedish Landrace), 1–4 years old, housed at the Division of Clinical Sciences, Swedish University of Agricultural Sciences (SLU), Uppsala, Sweden. All boars were fed and housed according to Swedish husbandry standards [13] and were provided with water *ad libitum*. The experimental protocol had previously been reviewed and approved by the Ethical Committee for Experimentation with Animals, Uppsala, Sweden. 

### 2.1. Semen Collection

The sperm-rich fraction (SRF) of ejaculates was collected using the gloved-hand technique into a plastic bag in a prewarmed thermos flask. The semen was immediately extended 1 : 1 (v/v) in warm (35°C) Beltsville Thawing Solution (BTS; [14]).

### 2.2. Media

BTS consisted of 200 mL deionised water, 7.4 g glucose, 1.2 g trisodium citrate, 0.26 g sodium bicarbonate, 0.26 g sodium ethylene diamine tetraacetic acid (EDTA), and 0.16 g potassium chloride. The BTS was prepared on the day of semen collection. 

Androcoll-P and Androcoll-P-Large (SLU, Uppsala, Sweden) are a ready-to-use glycidoxypropyltrimethoxysilane-coated silica colloid in a species-specific buffered salt solution. No preparation is required other than equilibration to room temperature (22–23°C) before use.

### 2.3. Sperm Concentration

Sperm concentration was measured using a Nucleocounter-SP100 (Chemometec, Allerød, Denmark), according to the manufacturer's instructions [15]. The sperm concentration in the extended ejaculates was adjusted to 100 × 10^6^ spermatozoa/mL with BTS for SLC. The sperm concentration in the SLC-selected samples was also measured, to allow the yield obtained to be calculated.

### 2.4. Single Layer Centrifugation

Aliquots of the extended semen were layered on top of a single layer of Androcoll-P, using different volumes according to the experimental design (see below).

After centrifuging at 300 g for 20 min, the resulting sperm pellets were resuspended in BTS containing either 1.25 mg/mL bovine serum albumin (BSA; Bovine Serum Albumin Fraction V, Sigma, Stockholm) (Experiment 1) or 5.0 mg/mL BSA (Experiment 2). All sperm samples were subsequently stored in a Climate Box (Unitron, Tørring, Denmark) at a temperature of 16 to 18°C. Aliquots of unselected and SLC-selected samples were taken for various analyses as described below.

### 2.5. Computer-Assisted Sperm Motility Analysis (CASA)

Sperm motility in all samples was assessed after incubating in a water bath at 38°C for 30 min. An aliquot (5 *μ*L) of the sample in a prewarmed Makler counting chamber (Sefi Medical Instruments, Haifa, Israel) was placed on the warm (38°C) microscope stage of a phase-contrast microscope and analyzed by CASA using a Mika Cell Motion Analyser (CMA) (MTM Medical Technologies Montreux, Switzerland). The software settings, which had been previously determined to be optimal [16], were as follows: 32 frames per sequence, a minimum of 15 frames per object, minimum area for objects = 25 pix, velocity limit for immobile objects = 10 *μ*m/s, and velocity limit for locally motile objects = 25 *μ*m/s. Motile spermatozoa were classified in three categories: linear (spermatozoa deviating less than 10% from a straight line), circular (spermatozoa moving in a circle with a radius less than 25 *μ*m), and nonlinear, the latter being spermatozoa falling into neither of the first two categories [16]. At least 200 spermatozoa were analyzed for each sample. The software reports proportions of immotile, locally motile, and motile spermatozoa, with the latter being further classified as circular, nonlinear, and linear motile populations, in addition to other parameters. For the purposes of this report, only the proportion of motile spermatozoa and the three subclasses (circular, nonlinear, and linear motile) were considered for analysis.

### 2.6. Morphology

Aliquots for morphology analysis were taken on Day 0. The methods have been described in detail previously [8]. Briefly, aliquots of the fresh extended ejaculates and centrifuged sperm preparations were used to prepare air-dried slides for assessment of sperm head shape of 500 spermatozoa at X1000 magnification. The following abnormalities were assessed: proximal cytoplasmic droplets, detached heads, acrosome defects, nuclear pouches, midpiece defects, tail defects (bent, coiled, or double bend). The proportions were used to calculate the total pathological heads and the number of morphologically normal spermatozoa. In addition, aliquots were fixed in formol-saline for counting of 200 spermatozoa on wet smears (X1000) [17], focussing on the following: pear-shaped heads, and heads narrow at the base, heads with abnormal contour, undeveloped heads, detached heads, narrow (tapering) heads, heads of variable size. The mean proportion of morphologically normal spermatozoa was estimated as the remaining proportion left from total abnormal spermatozoa, including those spermatozoa with distal cytoplasmic droplets which were considered to be normal. Since the number of specific abnormalities was low in most cases (<1%), they have been classified as head and tail abnormalities in this study. As the total incidence of cytoplasmic droplets and midpiece defects was greater than 5% in the uncentrifuged ejaculates, these two types were considered separately from the other abnormalities. Morphology evaluation was carried out by skilled personnel according to the standard protocol in the Swedish Sperm Reference Laboratory at SLU.

### 2.7. Plasma Membrane Integrity

Aliquots from the unselected and selected samples were extended to a concentration of approximately 5 × 10^6^ sperm cells/ mL using BTS. Each sample was evaluated using a BD LSR flow cytometer (Beckon Dickinson, San José, CA, USA). Excitation was with an argon-ion laser (488 nm). Green fluorescence was detected with a FL1 band-pass filter (530/30 nm) while red fluorescence was measured using a FL3 long-pass filter (>670 nm). A total of 50,000 sperm-specific events were evaluated and calculated as percentages.

Assessment of plasma membrane integrity (PMI) was made using the procedure described by Johannisson et al. [18]. Briefly, 1000 *μ*L of each sample was stained with 1 *μ*L of SYBR-14 (final stain concentration 0.02 *μ*M) followed by 5 *μ*L of PI (final stain concentration 12 *μ*M) (Live-Dead Sperm Viability Lit L-7011; Invitrogen, Eugene, OR, USA). After incubating at 38°C for 10 minutes, the fluorescence was measured using the FC. The cells were classified as: living (SYBR14-positive/PI-negative), dead (SYBR14-negative/PI-positive), or dying (SYBR14-positive/PI-positive).

### 2.8. Reactive Oxygen Species (ROS)

**Figure 1:**
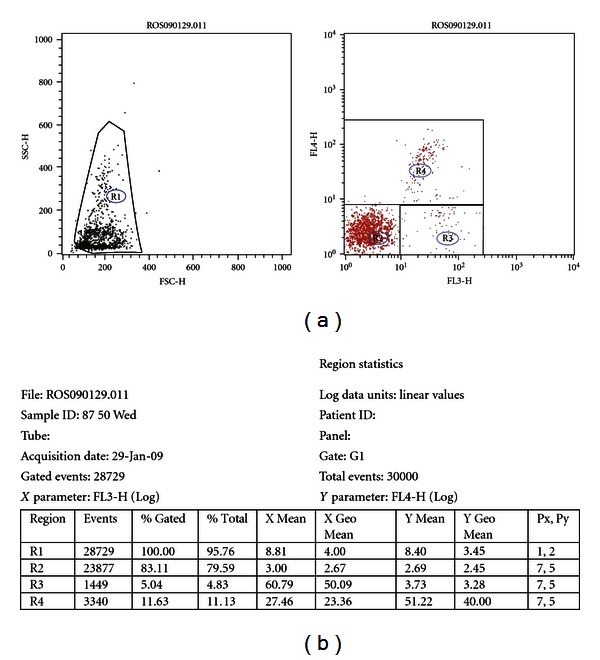
Flow cytometric analysis of boar spermatozoa stained with hydroethidine and dichlorodihydrofluorescein diacetate. Note: (left) R1 = total gated; (right) R2 = ROS-negative living; R3 = ROS-positive living; R4 = dead.

Aliquots from the unselected and selected samples were extended to a concentration of approximately 5 × 10^6^ sperm cells/mL using BTS and were stained as follows: an aliquot of 300 *μ*L from the extended samples was mixed with 3 *μ*L of Hoechst 33258 (HO) (final stain concentration 0.4 *μ*M), 3 *μ*L of hydroethidine (HE) (final stain concentration 0,4 *μ*M), and 3 *μ*L of dichlorodihydrofluorescein diacetate (DCFDA) (final stain concentration 20 *μ*M). HO was purchased from Sigma, Stockholm, while HE and DCFDA where purchased from Invitrogen Molecular Probes, Eugene, OR, USA. The samples were incubated at 38°C for 30 minutes before being analyzed by flow cytometry (FC). The method is a modification of that described previously [19], the modification being the use of HO as an independent analysis of living spermatozoa. Each sample was evaluated using a BD LSR flow cytometer. Excitation was with an argon-ion laser (488 nm) and a HeCd laser (325 nm). Detection of green fluorescence was with a FL1 band pass filter (530/30 nm), red fluorescence was measured using a FL3 long-pass filter (>670 nm), and blue fluorescence was detected in FL4 with a band-pass filter (510/20 nm). A total of 30,000 sperm-specific events were evaluated and calculated as percentages. The cells were classified as ROS-negative living, ROS-positive living, or dead in the HE-HO dotplot, after gating for sperm cells in the FSC-SSC-dotplot (Figure 1). The DCFDA fluorescence was not used for evaluation, since it was always highly correlated with the HE fluorescence.

### 2.9. Experimental Design

**Experiment 1**

Ejaculates (*n* = 5) were obtained from each of four boars (total sample size *n* = 20). A comparison was made of two volumes: SLC-4.5: 4 mL of Androcoll-P plus 4.5 mL of extended semen in a 12-mL conical centrifuge tube (12 mL centrifuge tube; Sarstedt, Landskrona, Sweden) and SLC-15: 15 mL of Androcoll-P-Large plus 15 mL semen in a 50-mL Falcon centrifuge tube. The control samples consisted of aliquots of uncentrifuged extended semen. Aliquots from three ejaculates per boar were taken for morphology evaluation and analysis of viability and ROS production on the day of semen collection (Day 0), whereas aliquots from all sperm samples were used for sperm motility assessment on Day 0, after 24 h storage (Day 1) and 72 h storage (Day 3) at 16–18°C.

**Experiment 2**

Ejaculates (*n* = 3 per boar) were collected from two of the boars from Experiment 1 and from other two (total sample size *n* = 12). For this experiment, SLC-4.5 (as above) was compared with SLC-25 (20 mL Androcoll-P plus 25 mL extended semen in a 100-mL glass centrifuge tube). The control samples were as described above. Morphology and motility evaluations were carried out as for Experiment 1.

### 2.10. Statistical Analysis

The statistical analyses were performed using the SAS software (Ver. 9, SAS Institute Inc., Cary, NC, USA). Variables were analyzed using analysis of variance (PROC MIXED). The statistical model included the fixed effects: boars, treatments (three: 4.5, 15, UN for Experiment 1; 4.5, 25, UN for Experiment 2) and the interaction between treatment and boar. Least squares means were calculated for each level of the fixed effect, and levels of significance were estimated for differences between least squares means.

## 3. Results

### 3.1. Sperm Motility

**Figure 2:**
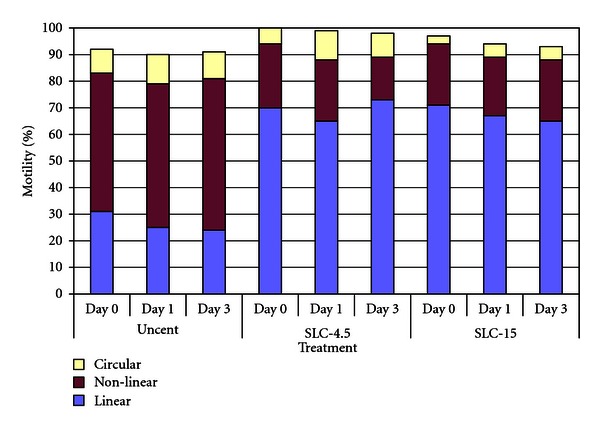
Motility patterns in SLC-selected spermatozoa (SLC-4.5 and SLC-25) and nonselected controls, showing the increase in linear motility and concomitant decrease in nonlinear motility in the SLC samples (*n* = 20).

**Figure 3:**
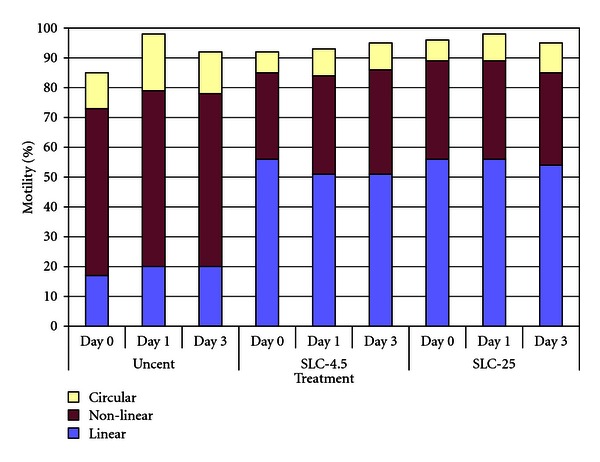
Motility patterns in SLC-selected spermatozoa (SLC-4.5 and SLC-25) and nonselected controls, showing the increase in linear motility and concomitant decrease in nonlinear motility in the SLC samples (*n* = 12).

In both experiments, there were no significant differences among treatments for total motility (Table 1), although there were significant differences in the proportions of linear and nonlinear motility between treatments and time, as shown in Figures 2 and 3 for Experiments 1 and 2, respectively, (*P* < .001). The variation between boars was not significant.

### 3.2. Yield after SLC

There was no difference in the mean yields for the different SLC treatments in either experiment (Experiment 1: SLC-4.5 66.5 ± 18%; SLC-15 58 ± 19%, NS; Experiment 2: SLC-4.5 40 ± 16%; SLC-25 39 ± 20%, NS). There was significant within-boar variation (*P* < .001).

### 3.3. Sperm Morphology

In both experiments, the proportion of morphologically normal spermatozoa (Tables 2 and 3) was significantly higher in the SLC samples than in the untreated samples (*P* < .001 in both experiments). There were fewer tail defects in the SLC-selected samples compared to the nonselected controls (*P* < .01 and *P* < .001 for Experiments 1 and 2, resp.), while in Experiment 2 there was also a trend towards fewer total head abnormalities in the SLC-selected samples compared to the nonselected controls (*P* < .054). There were no differences for other morphological abnormalities, which appeared with a prevalence of less than 1%, apart from proximal drops, and have not been included in the statistical analysis. Variation between boars was not significant.

### 3.4. Sperm Membrane Integrity

Mean values (±SD) for living, dying, and dead spermatozoa for the different treatments in Experiment 1 are shown in Table 4. There were no significant differences between the unselected and SLC-selected samples or between the two SLC treatments.

### 3.5. ROS Production

Table 4 shows mean (±SD) sperm viability measured with Hoechst 33258 in the ROS assay compared with the overall means for living spermatozoa from the SYBR14/PI staining. There was a difference in % living spermatozoa in all treatments between the ROS-staining method and the SYBR14-PI-staining method, with % living being higher in the SYBR14/PI assay. Regression analysis showed only a weak relationship between the two methods with a trend towards significance (*R* ^2^ = 0.312; *P* < .058). There were fewer ROS-positive spermatozoa and fewer dead spermatozoa in the unselected samples than in the selected samples (*P* < .01), although there were significant differences between boars (*P* < .001). There were more ROS-negative spermatozoa in the uncentrifuged samples than in both SLC 4.5 and SLC 15 mL samples (*P* < .01 for uncentrifuged versus SLC 4.5 mL, *P* < .001 for uncentrifuged versus SLC 15 mL).

## 4. Discussion

The objective of this paper was to scale up the original SLC method to allow larger volumes of ejaculate to be processed. The parameters of sperm quality assessed were sperm motility, morphology, membrane integrity and ROS production for Experiment 1 (scaling up to 4.5 ml and 15 mL), whereas in Experiment 2 (scaling up to 25 mL) only sperm motility and morphology were evaluated.

The proportions of motile spermatozoa were not different between the different treatments, in contrast to previous results where subjective motility assessment was used [11] However, in the present study, less BSA (1.25 mg/mL) was added to the BTS in Experiment 1 compared to previous studies, which resulted in spermatozoa adhering to the microscope slide, thus not being included in the CASA motility analysis, and also might have contributed to decreased sperm survival. Furthermore, the boars were older in the experiment reported here than in the previous study [11] which might have affected sperm quality. In Experiment 2 in the present study, 5.0 mg/mL BSA was added to the BTS, that is, the same concentration as was added in the previously reported study [11] which might have improved sperm survival in the SLC-selected samples, although some sperm aggregation was observed as a result. Again, these aggregated sperm clumps were ignored by the CASA analysis. Therefore, on the basis of these observations, it would appear that CASA is not helpful for analyzing motility in SLC-selected samples if BTS is used to resuspend the sperm pellets. 

Although the proportion of motile spermatozoa was not different between treatments, the motility patterns did differ, with SLC-selected spermatozoa showing more linear motility and less nonlinear motility than the unselected controls (Figures 2 and 3). The difference was less marked for the SLC-4.5 and SLC-25 treatments in Experiment 2 than that in Experiment 1, which might have been due to the increased content of BSA in the BTS, but nevertheless was significant (*P* < .001).

Normal morphology was significantly improved in all the SLC treatments compared to the nonselected controls. There was no difference between the different SLC-treatments for these parameters, or for sperm yield, indicating that scaling up the SLC method did not have a detrimental effect on sperm quality. These findings are consistent with previous observations made on stallion spermatozoa [9, 10] and preliminary observations on boar spermatozoa [11].

SLC treatment did not improve boar sperm viability, according to the SYBR-14/PI results (which was why it was excluded from Experiment 2). This observation is in contrast to results with stallion spermatozoa where viability was improved by SLC selection [20]. One explanation for this finding could be that the viability in the samples was already high and, therefore, hard to improve significantly. This result is in agreement with previous work on bull spermatozoa where there was no change in viability after density gradient centrifugation (DGC) where semen from animals of high fertility was used, although there was an improvement when semen from bulls of low fertility was subjected to DGC [21]. Previously, values for “living” spermatozoa determined by SYBR14-PI staining were 80%–90% for boars [22, 23]. In the study reported here, a higher percentage of living spermatozoa (up to 95%) were found, possibly because the concentration of the stain and/or the concentration of protein in the extender were not optimal. The SYBR14/PI staining method has been validated previously for bull spermatozoa [24]; similar staining patterns were subsequently found for the spermatozoa of other species [23]. 

The observation that there was less ROS production in the untreated samples than in the SLC-selected samples was, at first, surprising, since DGC has been shown to reduce ROS production in boar sperm samples. Previously, a concentration of less than 4% of ROS production was found [19], while in the current study it was around 6%. However, the SLC samples apparently contained a higher proportion of dead spermatozoa than in the non-SLC-selected samples, which might have been a source of ROS. Furthermore, different proportions of living spermatozoa were detected for the ROS staining method and the SYBR14-PI staining method, despite the analyses being performed on the same day, indicating that one of the staining procedures was not optimal for boar semen. Alternatively, the different incubation times for stained samples for the two techniques (10 min for SYBR/PI versus 30 min for HO/HE/DCFDA) might have contributed to the difference. Since the uncentrifuged samples contained seminal plasma, some ROS might have been removed by antioxidants present in the seminal plasma whereas no seminal plasma, and therefore no antioxidant activity were present in the SLC-samples. Previously, it has been suggested that some ROS production is necessary to obtain fertilization in IVF. In a preliminary IVF experiment with one of the boars, the oocytes fertilized with SLC-selected spermatozoa showed a 35% rate of development to blastocysts, compared with 19% for the non-SLC-selected sample (Gonzalez Herrero et al., unpublished observations).

One parameter of sperm quality not evaluated in this study is chromatin integrity. Previous experiments with stallion spermatozoa showed that SLC selects spermatozoa with good chromatin integrity [10]. However, unlike stallions, boars generally have very good chromatin integrity in the unselected ejaculate [25]. SCSA was done on ejaculates from the four boars used in Experiment 1 on another occasion: although the mean DFI values were 2 ± 1% for uncentrifuged and 0.7 ± 0.6% for SLC-selected spermatozoa, these values lie within the interassay variation for our flow cytometer and therefore do not necessarily represent selection for spermatozoa with good chromatin integrity (Morrell & Johannisson, unpublished data).

For most of the parameters measured, there were no significant differences between either the 4.5 mL-SLC and 15 mL SLC-treatments, or the SLC-4.5 and SLC-25 treatments indicating that the SLC can be scaled up to 4.5 mL, 15 mL, or 25 mL without reducing sperm quality in the resulting samples. These results confirm earlier observations with stallion spermatozoa [11], where 15 mL extended semen was used on 15 mL Androcoll-E-Large. The SLC-25 protocol would be feasible for producing AI doses from very valuable boars since approximately 1500 × 10^6^ spermatozoa could be obtained per SLC-25, that is, half a normal AI dose. However, the relevance of the technique would depend on the boar: when the SLU boars were compared with Hampshire boars on a commercial boar station, there were considerable among group differences for all parameters of sperm quality (*P* < .001; data not shown). Further adjustments are required to scale up the technique to a larger volume and to investigate among boar variation. However, overall these results are very promising in that it seems to be possible to improve some aspects of sperm quality, such as normal morphology and progressive motility, in boar semen samples with SLC and that sufficient sperm numbers could be obtained for AI doses with the larger volumes from some boars. SLC-selected spermatozoa showed normal functionality in the IVF study referred to previously, which is in agreement with fertility data from bulls [26] and stallions [27], respectively. 

In conclusion, SLC can be used to enhance the quality of boar sperm samples, particularly for normal morphology and linear motility. Furthermore, the technique could be scaled-up to process 15 mL or 25 ml extended ejaculate per tube without compromising *in vitro* assessed sperm quality in the selected samples. These results are encouraging as preliminary steps to a further scale up procedure, to enable large volumes of ejaculate to be processed for the swine insemination industry.

## Figures and Tables

**Table 1:** Mean (±SD) motility in boar sperm samples with and without single layer centrifugation through Androcoll-P in SLC-4.5 and SLC-15 (*n* = 20).

	Uncent. (%)	SLC-4.5 (%)	SLC-15 (%)
Day 0	75 ± 16	78 ± 11	73 ± 17
Day 1	66 ± 21	63 ± 19	65 ± 19
Day 3	67 ± 21	47 ± 24	47 ± 13

**Table 2:** Percentage of spermatozoa with normal morphology (%) (mean ± SD) in boar sperm samples before and after Single layer centrifugation (*n* = 12 ejaculates).

Group	Unselected (%)	SLC-4.5 (%)	SLC-15 (%)
Normal morphology	83.6 ± 13.6^a^	96.5 ± 3.6^a^	94.0 ± 5.1^a^
Abnormal heads	4 ± 0.9	4 ± 1.1	4 ± 1.2
Tail defects	9.4 ± 10.9^bc^	0.4 ± 0.5^b^	1.6 ± 2.6^c^

Note: superscript letter denotes significant difference in the SLC samples than in the non-SLC-selected samples, ^a^ *P* < .001, ^b^ *P* < .01, ^c^ *P* < .05.

**Table 3:** Effect of Single layer centrifugation on boar sperm morphology in Experiment 2 (*n* = 12).

	Uncent. (%)	SLC-4.5 (%)	SLC-25 (%)
Normal morphology	82 ± 14^ab^	97 ± 1.4^a^	97 ± 2^b^
Abnormal heads	3.15 ± 1.3^c^	2.3 ± 0.8^c^	2.3 ± 0.7^c^
Abnormal tails	7.0 ± 10.4^c^	0.2 ± 0.2^c^	0.3 ± 0.5^c^

Note: superscript letter denotes significant difference in the SLC samples than in the non-SLC-selected (uncentrifuged) samples, ^a^ *P* < .001, ^b^ *P* < .01, ^c^ *P* < .05.

**Table 4:** Proportion (%) (mean ± SD) of living, ROS producing, dead, and dying boar spermatozoa measured 24 h after semen collection and SLC (*n* = 16 ejaculates).

Parameter	Classification	Unselected (%)	SLC-4.5 (%)	SLC-15 (%)
	Living	94 ± 2.7	92.8 ± 3.2	94.4 ± 2.5
SYBR14/PI	Dying	4.7 ± 2.3	5.6 ± 2.4	4.2 ± 1.8
	Dead	1.3 ± 0.6	1.4 ± 0.7	1.2 ± 0.8

	Living ROS −ve	85.1 ± 3.6^ac^	67.9 ± 13.1^a^	72.1 ± 16.6^c^
ROS	Living ROS +ve	4.8 ± 1.7^bc^	8.4 ± 3.5^b^	8.6 ± 5.7^c^
	Dead	9.6 ± 2.6^a^	23.1 ± 9.5^a^	18.9 ± 10.9^b^

Note: ROS = reactive oxygen species; SYBR14/PI staining for membrane integrity. ^a^ *P* < .001; ^b^ *P* < .01; ^c^ *P* < .05.

For viability (SYBR14/PI), there were no statistically significant differences among treatments.
